# Expression of Concern: Comparative proteomic analysis reveals the regulatory network of the veA gene during asexual and sexual spore development of *Aspergillus cristatus*

**DOI:** 10.1042/BSR-20180067_EOC

**Published:** 2021-03-31

**Keywords:** ascospore, Aspergillus cristatus, conidia, proteomics, veA gene

The Editorial Office has been made aware of potential issues surrounding the scientific validity of this paper, hence has issued an expression of concern to notify readers whilst the Editorial Office investigates.

